# National-level assessment of gestational carrier pregnancies in the United States

**DOI:** 10.1007/s10815-024-03320-5

**Published:** 2024-11-20

**Authors:** Aaron D. Masjedi, Rachel S. Mandelbaum, Katherine V. Erickson, Zachary S. Anderson, Shinya Matsuzaki, Joseph G. Ouzounian, Koji Matsuo, Richard J. Paulson

**Affiliations:** 1 Division of Gynecologic Oncology, Department of Obstetrics and Gynecology, University of Southern California, 2020 Zonal Avenue, IRD520, Los Angeles, CA 90033 USA; 2 Division of Reproductive Endocrinology and Infertility, Department of Obstetrics and Gynecology, University of Southern California, Los Angeles, CA USA; 3 Keck School of Medicine, University of Southern California, Los Angeles, CA USA; 4 Department of Obstetrics and Gynecology, Osaka University Graduate School of Medicine, Osaka, Japan; 5 Division of Maternal-Fetal Medicine, Department of Obstetrics and Gynecology, University of Southern California, Los Angeles, CA USA; 6 Norris Comprehensive Cancer Center, University of Southern California, Los Angeles, CA USA

**Keywords:** Gestational carrier, Pregnancy, Trends, Characteristics, Maternal morbidity

## Abstract

**Purpose:**

To assess national trends, characteristics, and delivery outcomes associated with gestational carriers (GC) pregnancies.

**Methods:**

This cross-sectional study queried the Healthcare Cost and Utilization Project’s National Inpatient Sample. The study population was 14,312,619 deliveries between 2017 and 2020. Obstetric characteristics and outcomes associated with GC pregnancies were assessed with inverse probability of treatment weighting propensity score.

**Results:**

There were 1965 GCs (13.7 per 100,000) included for national estimates. The prevalence rate of GC pregnancies increased by 55.0% over a 4-year period from 11.8 to 18.2 per 100,000 deliveries (*P*-trend < .001). In the weighted model, GCs were more likely to have a multiple gestation pregnancy (14.7% vs 1.8%, adjusted odds ratio [aOR] 7.83, 95% confidence interval [CI] 6.54–9.38, *P* < .001), placental abruption (3.5% vs 1.1%, aOR 2.98, 95%CI 2.12–4.19), and low-lying placenta (1.6% vs 0.2%, aOR 5.14, 95%CI 3.10–8.52). Among singleton delivery, odds of late-preterm (10.8% vs 6.4%, aOR 1.79, 95%CI 1.44–2.23) and periviable (1.1% vs 0.4%, aOR 2.54, 95%CI 1.32–4.89) deliveries and postpartum hemorrhage (12.2% vs 4.1%, aOR 3.27, 95%CI 2.67–4.00) were increased for GC compared to non-GCs whereas odds of cesarean delivery (23.6% vs 31.6%, aOR 0.59, 95%CI 0.51–0.69) were decreased. These associations were less robust in multi-fetal gestations.

**Conclusion:**

The results of the current nationwide assessment suggest that GC pregnancies are rare but gradually increasing in the United States. This study shows that GC pregnancies have usually favorable pre-pregnancy patient characteristics compared to non-GC pregnancies, with mixed obstetric outcomes including increased odds of preterm delivery, placental abnormalities, and postpartum hemorrhage and decreased odds of cesarean delivery in singleton pregnancies.

**Supplementary Information:**

The online version contains supplementary material available at 10.1007/s10815-024-03320-5.

## Introduction

A gestational carrier (GC) is an individual who gestates a genetically unrelated child or children on behalf of the intended parent(s) via in-vitro fertilization (IVF). Common indications for seeking a GC include male same-sex couples, repeated autologous IVF failures or recurrent miscarriages, and maternal conditions that preclude or confer higher than acceptable risk in pregnancy, among others [1]. GCs are generally selected based on favorable characteristics for pregnancy, both to minimize obstetric risk for the GC and to maximize the chances of a healthy child for the intended parent(s).

GC pregnancies have been steadily increasing since the first GC pregnancy in 1985 [2–4]. In 2011, there were 2841 total cycles involving a GC in the U.S.; this statistic more than tripled to 9195 in 2019 [5]. Despite the rapid spike in GC utilization, national data on outcomes and risks associated with GC pregnancies are lacking. Some literature suggests that obstetric risks are decreased compared to the general population given GCs are a preselected low-risk cohort, while other studies have highlighted specific risks such as that of preterm birth [3, 6–9]. In recent years, the Society for Assisted Reproductive Technology and the American Society for Reproductive Medicine (ASRM) have focused on rates of multiple gestation in GC pregnancies as a quality metric and have called out programs that are outliers [1, 10]. The focus on this outcome and the official recommendation for single embryo transfers likely impacts outcomes in GC pregnancies given the known risks associated with multifetal gestations.

This information is particularly important for two main reasons. First, consent for a GC is a highly complex process, both ethically and legally. It is crucial to have a complete understanding of the antenatal, intrapartum, and postpartum risks involved in a GC pregnancy. These risks can then be discussed with potential GCs so true informed consent may be obtained. Second, studying outcomes of GC pregnancies is an opportunity to examine the risks involved with the IVF process without the confounders of infertility.

The objective of this study was to examine national trends, characteristics, and delivery outcomes associated with GC pregnancies as compared to non-GC pregnancies.

## Materials and methods

### Data source

This cross-sectional study queried the Healthcare Cost and Utilization Project’s National Inpatient Sample [11]. The Healthcare Cost and Utilization Project is the United States health service data platform that is supported by the Agency for Healthcare Research and Quality, one of the twelve federal agencies within the United States Department of Health and Human Service.

The National Inpatient Sample approximates a stratified sample of 20% of discharges in each center from all the participating hospitals across 48 states and the District of Columbia. Every year, the dataset captures more than 7 million inpatient admissions. In 2020, a total of 4580 hospitals participated in the program. In each encounter, the program captures a maximum of 40 diagnoses and 25 procedures during the index hospitalization. When weighted for national survey estimates, it covers more than 97% of the U.S. population. The University of Southern California Institutional Review Board exempted this study due to the use of publicly available, deidentified data.

### Study eligibility

The study population included patients who had a hospital delivery from 2017 to 2020. Identification of hospital delivery was based on the World Health Organization’s International Classification of Disease, 10th revision (ICD-10) Clinical Modification and Procedural Coding Schema codes and Disease-Related Group codes that followed prior investigations (Table S1) [12, 13]. Patient age was restricted to 15–54 years as per prior studies [13, 14]. The starting point of 2017 was chosen due to the introduction of the ICD-10 Clinical Modification code for GC.

### Exposure

The eligible cases were grouped based on the diagnosis of GC, identified according to the ICD-10 Clinical Modification code (Table S1). Specifically, patients who had the ICD-10 code for GC were classified as the GC group, and those who did not have the code were classified as the non-GC group.

### Outcome measures

Obstetric characteristics including pregnancy complications and delivery outcomes were evaluated as the main outcomes in this study. The ICD-10 Clinical Modification and Procedural Classification System codes were used to identify the measured outcomes that followed prior coding schemas (Table S1). [12, 13, 15]

Pregnancy factors evaluated included maternal factors (gestational diabetes, gestational hypertension, pre-eclampsia, excess weight gain during pregnancy), fetal factors (multifetal gestations including triplet pregnancy, intrauterine growth restriction, large for gestational age, fetal anomaly, fetal malpresentation, and intrauterine fetal demise), placental factors (placental abruption, placenta previa, low-lying placenta, placenta accreta spectrum, placental malformation, and vasa previa), membranous/fluid factors (premature rupture of membrane [preterm and term], chorioamnionitis, oligohydramnios, and polyhydramnios), umbilical cord factor (umbilical cord prolapse), and utero-cervical factors (uterine rupture and cervical insufficiency).

Delivery outcomes evaluated included gestational age at delivery, cesarean delivery, operative delivery, postpartum hemorrhage, blood product transfusion, and severe maternal morbidity (SMM). These measured indicators followed the Center for Disease Control and Prevention definition [16]: acute myocardial infarction, acute renal failure, adult respiratory distress syndrome, air and thrombotic embolism, amniotic fluid embolism, aneurysm, cardiac arrest/ventricular fibrillation, cardiac rhythm conversion, disseminated intravascular coagulation, eclampsia, heart failure/arrest during surgery or procedure, hysterectomy, puerperal cerebrovascular disorders, pulmonary edema/acute heart failure, severe anesthesia complications, sepsis, shock, sickle cell disease with crisis, temporary tracheostomy, and ventilation.

### Study variables

Baseline non-pregnancy demographics evaluated included patient age (< 25, 25–29, 30–34, 35–39, and ≥ 40 years), year (2017, 2018, 2019, and 2020), race and ethnicity (Asian, Black, Hispanic, Native American, Other, and White) determined by the program, primary payer (Medicaid, private insurance including health maintenance organization, self-pay, and other), census-level median household income (every quarter), patient location (large central metropolitan, large fringe metropolitan, medium metropolitan, small metropolitan, micropolitan, and not metropolitan or micropolitan counties), and housing status. Race and ethnicity were included in a view of relevance to pregnancy characteristics and outcomes.

Hospital parameters included regions of the United States (Northeast, Midwest, South, and West), facility relative bed capacity (small, mid, and large), and facility location and teaching status (rural, urban non-teaching, and teaching). These hospital parameters were determined by the program.

Medical comorbidities included obesity, pregestational hypertension, pregestational diabetes mellitus, and asthma. Gynecological factors included uterine factors (prior uterine scar, uterine myoma, uterine adenomyosis, uterine anomaly), endometriosis, cervical carcinoma in situ, and polycystic ovary syndrome. Substance factors included tobacco use, alcohol use, and illicit drug use. Mental health conditions included anxiety, depressive, bipolar, and schizophrenia disorders. Infectious disease factors included gonorrhea, syphilis, hepatitis virus, and anogenital herpes. Past pregnancy factors included grand multiparity and pregnancy loss.

### Statistical analysis

Prevalence rates of GC were aggregated in each year, and the temporal trend was assessed with the Cochran-Armitage test. Independent baseline demographics associated with GC compared to non-GC were assessed with a multivariable binary logistic regression model. Conditional backward selection was fitted due to the assumption of the rarity of GCs, to avoid overfitting [17]. Initial covariate selection was set as the *P* < 0.05 level in the univariable analysis. The least significant covariate was then removed from the model sequentially until all the covariates retained *P* < 0.05 in the final model. Multicollinearity was assessed among the study covariates. The effect size for GC vs non-GC was expressed with adjusted odds ratio (aOR) and a corresponding 95% confidence interval (CI).

Inverse probability of treatment weighting (IPTW) propensity scoring was used to mitigate the difference in baseline pre-pregnant demographics between the GC and non-GC groups [18]. Independent characteristics between the two exposure groups determined by the prior step analysis were considered to create the IPTW cohort. The IPTW propensity score method assigned patients in the GC group a weight of 1/(propensity score) and those in the non-GC group a weight of 1/(1-propensity score). Stabilized weights and threshold technique at 10 were used. Standardized difference between the two exposure groups was assessed, and a value of > 0.20 was informed for model adjusting. Pregnancy characteristics were then assessed in the ITPW cohort. Delivery outcomes were also assessed in the IPTW cohort, further adjusting for pregnancy confounders between the two exposure groups. This analytic approach was based on the rationale that pregnancy events chronologically followed pre-pregnant conditions.

Various sensitivity analyses were conducted to assess the robustness of the study findings. First, the temporal trend of the multifetal gestation rate was assessed among pregnant patients with GC labeling. Second, cesarean delivery rate was assessed among those who did not have a prior cesarean section. Third, the vaginal birth rate among the patients with a history of cesarean delivery was evaluated. Fourth, SMM was assessed per the extent of gestation or per past uterine scar status. Last, the study cohort was stratified by the number of fetuses (singleton or multi-fetal gestations).

All statistical analyses were based on two-tailed hypotheses, and a *P* < 0.05 was considered statistically significant. The weighted values for national estimates provided by the programs were used for the analysis. Missing values in each study covariate were grouped for analysis. Statistical Package for Social Sciences (IBM SPSS, version 28.0, Armonk, NY, USA) and R statistics (version 3.5.3, R foundation for Statistical Computing, Vienna, Austria) were used for the analysis. The STROBE guidelines were consulted for the performance of this study.

## Results

### Prevalence and trends of GC

**Fig. 1:**
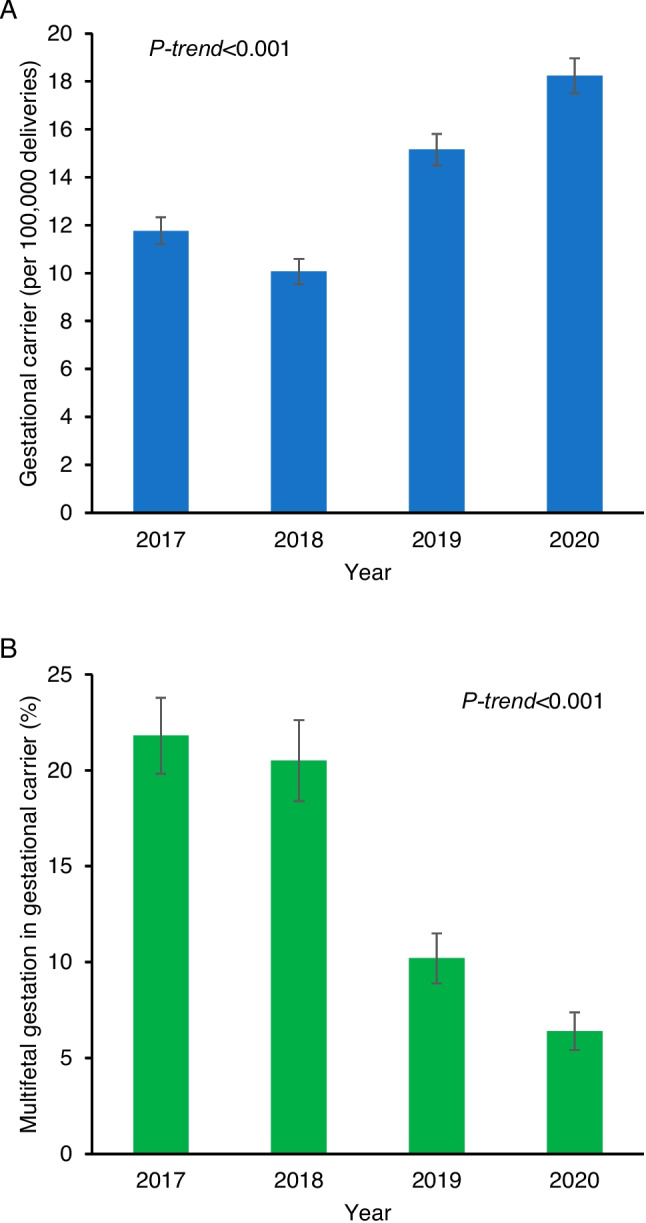
Temporal trends of gestational carrier pregnancies. **A** Prevalence rates of gestational carrier pregnancy per 100,000 hospital deliveries and **B** proportion of multifetal gestations among gestational carrier pregnancies are shown from 2017 to 2020. Observed values with standard error are displayed. Cochran-Armitage trend test for *P*-value

A total of 14,312,629 hospital deliveries were examined for the analysis. During the study period, there were 1965 GCs reported for the national estimates, with a prevalence rate of 13.7 per 100,000 hospital deliveries. In other words, one in 7284 hospital deliveries was for a GC. The prevalence rate of GC pregnancies increased by 55.0% over the study period from 11.8 to 18.2 per 100,000 deliveries (*P*-trend < 0.001; Fig. 1A). Among 1965 pregnant patients with GC, the prevalence of multifetal gestations decreased by 70.4% from 21.8% in 2017 to 6.4% in 2020 (*P*-trend < 0.001; Fig. 1B).

### Baseline characteristics related to GC

The median age of patients in the GC and non-GC groups was 33 (IQR 29–36) and 29 (IQR 25–33) years, respectively (*P* < 0.001). In a multivariable analysis (Table 1), pregnant patients in the GC group were more likely to be ≥ 35 years of age (35.9% vs 18.5%) including 35–40 years (28.8% vs 15.1%, aOR 1.57, 95%CI 1.37–1.77, *P* < 0.001) and ≥ 40 years (7.1% vs 3.4%, aOR 1.82, 95%CI 1.51–2.20, *P* < 0.001), White (70.0% vs 50.5%, *P* < 0.001), Western U.S. residents (60.1% vs 23.6%, aOR4.23, 95%CI 3.65–4.89, *P* < 0.001), and have private insurance (91.3% vs 51.7%, aOR 1.87, 95%CI 1.43–2.45, *P* < 0.001) compared to those in the non-GC group; pregnant patients in the GC were more likely to deliver at large (58.3% vs 50.3%, aOR 1.22, 95%CI 1.08–1.38, *P* = 0.001), urban non-teaching (22.9% vs 18.9%, aOR 1.18, 95%CI 1.06–1.31, *P* = 0.003) centers.

**Table 1:** Baseline demographics associated with gestational carrier

Characteristic	Non-GC	GC	aOR (95%CI)^*§*^	*P*-value
No	14,310,664 (100)	1965 (100)		
**Age (y)**	29 (25–33)	33 (29–36)		< 0.001*
< 25	3,413,419 (23.8)	100 (5.1)	0.35 (0.28–0.44)	< 0.001
25–29	4,120,753 (28.7)	520 (26.5)	1.00 (reference)	
30–34	4,139,833 (28.9)	640 (32.6)	0.97 (0.86–1.09)	0.560
35–40	2,156,379 (15.1)	565 (28.8)	1.57 (1.39–1.77)	< 0.001
≥ 40	482,245 (3.4)	140 (7.1)	1.82 (1.51–2.20)	< 0.001
**Race/ethnicity**				< 0.001*
Asian^†^	855,309 (6.0)	35 (1.8)	0.11 (0.08–0.15)	< 0.001
Black	2,087,420 (14.6)	150 (7.6)	1.11 (0.94–1.32)	0.233
Hispanic	2,886,749 (20.2)	335 (17.0)	0.81 (0.72–0.92)	0.001
Native American	100,740 (0.7)	**	0.79 (0.42–1.47)	0.459
Other	630,885 (4.4)	30 (1.5)	0.32 (0.22–0.46)	< 0.001
Unknown	516,849 (3.6)	**	0.29 (0.20–0.42)	< 0.001
White	7,232,712 (50.5)	1375 (70.0)	1.00 (reference)	
**Primary payer**				< 0.001*
Medicaid	6,038,683 (42.2)	80 (4.1)	0.14 (0.10–0.19)	< 0.001
Private including HMO	7,401,921 (51.7)	1795 (91.3)	1.87 (1.43–2.45)	< 0.001
Self-pay	365.930 (2.6)	35 (1.8)	0.96 (0.63–1.47)	0.863
Other	488,805 (3.4)	55 (2.8)	1.00 (reference)	
Unknown	17,290 (0.1)	0	n/a	
**Hospital bed capacity**				< 0.001*
Small	2,824,124 (19.7)	330 (16.8)	1.00 (reference)	
Mid	4,292,951 (30.0)	490 (24.9)	1.02 (0.89–1.18)	0.749
Large	7,193,589 (50.3)	1145 (58.3)	1.22 (1.08–1.38)	0.001
**Hospital location/teaching**				0.012*
Rural	1,284,008 (9.0)	130 (6.6)	1.07 (0.89–1.28)	0.480
Urban non-teaching	2,701,001 (18.9)	450 (22.9)	1.18 (1.06–1.31)	0.003
Urban teaching	10,325,656 (72.2)	1385 (70.5)	1.00 (reference)	
**Region**				< 0.001*
Northeast	2,283,209 (15.9)	220 (11.2)	1.00 (reference)	
Midwest	3,014,888 (21.1)	305 (15.5)	1.04 (0.87–1.24)	0.650
South	5,638,932 (39.4)	260 (13.2)	0.55 (0.46–0.66)	< 0.001
West	3,373,635 (23.6)	1180 (60.1)	4.23 (3.65–4.89)	< 0.001
**Chronic hypertension**				
No	13,902,604 (97.1)	1940 (98.7)	1.00 (reference)	
Yes	408,085 (2.9)	25 (1.3)	0.50 (0.33–0.74)	< 0.001
**Obesity**				
No	12,582,660 (87.9)	1840 (93.6)	1.00 (reference)	
Yes	1,728,005 (12.1)	125 (6.4)	0.52 (0.44–0.63)	< 0.001
**Depression**				
No	13,751,930 (96.1)	1905 (96.9)	1.00 (reference)	
Yes	560,640 (3.9)	60 (3.1)	0.76 (0.58–0.98)	0.033
**Tobacco use**				
No	13,573,790 (94.9)	1950 (99.2)	1.00 (reference)	
Yes	736,875 (5.1)	15 (0.8)	0.45 (0.27–0.75)	0.002
**Drug use**				
No	13,906,009 (97.2)	**	1.00 (reference)	
Yes	404,655 (2.8)	**	0.31 (0.13–0.76)	0.010

Percentage per column or median (IQR) is shown. ^§^Multivariable binary logistic regression model with conditional backward selection. The final model is displayed. ^†^Including Pacific Islanders. *Overall *P*-value. **A small number of related cells are suppressed per HCUP guidelines. Abbreviations: *GC*, gestational carrier; *aOR*, adjusted odds ratio; *CI*, confidence interval

Contrarily, pregnant patients in the GC group were less likely to be < 25 years (5.1% vs 23.8%, aOR 0.35, 95%CI 0.28–0.44, *P* < 0.001), Asian (1.8% vs 6.0%, aOR 0.11, 95%CI 0.08–0.15, *P* < 0.001), obese (6.4% vs 12.1%, aOR 0.52, 95%CI 0.44–0.63, *P* < 0.001), hypertensive (1.3% vs 2.9%, aOR 0.50, 95%CI 0.33–0.74, *P* < 0.001), tobacco users (0.8% vs 5.1%, aOR 0.45, 95%CI 0.27–0.75, *P* = 0.002) compared to those in the non-GC group in the multivariable analysis (Table 1).

### Pregnancy characteristics related to GC

The IPTW cohort was created by modeling 12 independent factors that differed between the GC and non-GC groups as shown in Table 1. The difference in these modeled baseline characteristics was more balanced between the two groups in the ITPW cohort (Table S2).

In a multivariable analysis (Table 2), pregnant patients in the GC group were more likely to have a multiple gestation pregnancy (14.7% vs 1.8%, aOR 7.83, 95%CI 6.54–9.38), placental abruption (3.5% vs 1.1%, aOR 2.98, 95%CI 2.12–4.19), low-lying placenta (1.6% vs 0.2%, aOR 5.14, 95%CI 3.10–8.52), and preterm premature rupture of membranes (5.4% vs 2.7%, aOR 1.35, 95%CI 1.02–1.79) compared to those in the non-GC group. Contrarily, pregnant patients in the GC group were less likely to have polyhydramnios (aOR 0.15, 95%CI 0.05–0.46) and chorioamnionitis (0.9% vs 2.4%, aOR 0.38, 95%CI 0.20–0.76).

**Table 2:** Pregnancy characteristics associated with gestational carrier

Characteristic	non-GC	GC	aOR (95%CI)	*P*-value
**Multifetal gestation**				
No	98.2	85.3	1.00 (reference)	
Yes	1.8	14.7	7.83 (6.54–9.38)	< 0.003
**Placenta abruption**				
No	98.9	96.5	1.00 (reference)	
Yes	1.1	3.5	2.98 (2.12–4.19)	< 0.001
**Low-lying placenta**				
No	99.8	98.4	1.00 (reference)	
Yes	0.2	1.6	5.14 (3.10–8.52)	< 0.002
**Polyhydramnios**				
No	98.4	**	1.00 (reference)	
Yes	1.6	**	0.15 (0.05–0.46)	0.004
**Placental malformation**				
No	99.2	97.0	1.00 (reference)	
Yes	0.8	3.0	2.54 (1.77–3.66)	< 0.005
**Preterm PROM**				
No	97.3	94.6	1.00 (reference)	
Yes	2.7	5.4	1.35 (1.02–1.79)	0.038
**Chorioamnionitis**				
No	97.6	99.1	1.00 (reference)	
Yes	2.4	0.9	0.38 (0.20–0.76)	0.005

Percentage per group is shown. Pregnancy characteristics were assessed in the inverse probability of treatment weighting cohort accounting for baseline non-pregnancy characteristics. A multivariable binary logistic regression model with a conditional backward selection method was used to assess independent pregnancy characteristics as shown in the table. Four non-pregnancy characteristics that exhibited a standardized difference of > 0.2 were informed in the modeling. **A small number of data were suppressed per HCUP guidelines. Abbreviations: *GC*, gestational carrier; *aOR*, adjusted odds ratio; *CI*, confidence interval; *PROM*, premature rupture of membrane

### Delivery outcomes related to GC

The measured delivery outcomes were assessed in the IPTW cohort (Table 3). The majority of the pregnant patients in the GC group had a term delivery (81.2%). However, pregnant patients in the GC group were more likely to have a late-preterm delivery (34–36^6/7^ weeks gestation, 13.2% vs 7.0%, aOR 1.31, 95%CI 1.06–1.63) compared to those in the non-GC group.

**Table 3:** Delivery outcomes associated with gestational carrier

Characteristic	non-GC	GC	aOR (95%CI)	*P*-value
**Gestational age (w)**				
≥ 39	61.3	52.2	1.00 (reference)	
37–38	27.3	29.0	1.11 (0.95–1.28)	0.184
34–36	7.0	13.2	1.31 (1.06–1.63)	0.014
26–33	2.4	4.2	1.00 (0.71–1.42)	0.983
22–25	0.5	1.0	1.28 (0.66–2.48)	0.466
< 22	0.3	0	n/a	
Unknown	1.3	0.4	0.34 (0.13–0.90)	0.029
**Cesarean delivery**				
No	67.7	69.6	1.00 (reference)	
Yes	32.3	30.4	0.92 (0.80–1.05)	0.198
**Primary cesarean delivery**				
No	79.7	81.1	1.00 (reference)	
Yes	20.3	18.9	0.92 (0.77–1.09)	0.330
**Vaginal birth after cesarean**				
No	87.0	87.0	1.00 (reference)	
Yes	13.0	13.0	0.98 (0.62–1.54)	0.931
**Operative delivery**^§^				
No	94.3	94.8	1.00 (reference)	
Yes	5.7	5.2	0.90 (0.64–1.26)	0.618
**Postpartum hemorrhage**				
No	95.8	87.6	1.00 (reference)	
Yes	4.2	12.4	2.72 (2.25–3.29)	< 0.001
**Blood transfusion**				
No	98.8	98.5	1.00 (reference)	
Yes	1.2	1.5	1.29 (0.77–2.14)	0.333
**Severe maternal morbidity**^†^				
No	98.2	98.4	1.00 (reference)	
Yes	1.8	1.6	0.90 (0.55–1.48)	0.796
**Hysterectomy**				
No	99.8	**	1.00 (reference)	
Yes	0.2	**	0.43 (0.06–3.08)	0.735

Percentage per group is shown. Delivery outcomes were assessed in the inverse probability of treatment weighting cohort accounting for baseline non-pregnancy characteristics. For gestational age at delivery and postpartum hemorrhage, the exposure-outcome was adjusted for four baseline characteristics exhibiting a standardized difference of > 0.20 shown in Table S2 and two pregnancy characteristics that differed between the two groups and historically known for delivery outcomes (placenta abruption and multifetal gestation). **A small number of data were suppressed per HCUP guidelines. ^§^Vaginal delivery. ^†^Including blood transfusion. Abbreviations: *GC*, gestational carrier; *aOR*, adjusted odds ratio; *CI*, confidence interval

GC pregnancies were also associated with a nearly threefold increased risk of postpartum hemorrhage at delivery compared to those without (12.4% vs 4.2%, aOR 2.72, 95%CI 2.25–3.29). Cesarean delivery rates were similar between the pregnant patients in the GC and non-GC groups (30.4% vs 32.3%, aOR 0.92, 95%CI 0.80–1.05), including primary cesarean delivery rate (18.9% vs 20.3%, aOR 0.92, 95%CI 0.77–1.09). The vaginal birth rate among patients with prior cesarean delivery was comparable in the two groups (13.0% vs 13.0%, aOR 0.98, 95%CI 0.62–1.54).

The SMM rates at delivery were also similar between the GC and non-GC groups (1.6% vs 1.8%, aOR 0.90, 95%CI 0.55–1.48), and the association remained consistent per gestation extent (singleton or multifetal gestations) or past uterine scar (no prior or past cesarean delivery) (all, *P* > 0.05). Among the GC group, multifetal gestations or past uterine scar were not associated with SMM (both, *P* > 0.05).

Delivery outcomes were assessed based on the number of fetuses (Table 4). Among singleton pregnancies, the risks of late-preterm delivery (10.8% vs 6.4%, aOR vs full-term delivery 1.79, 95%CI 1.44–2.23), periviable delivery (22–25^6/7^ weeks gestation, 1.1% vs 0.4%, aOR 2.54, 95%CI 1.32–4.89), and postpartum hemorrhage (12.2% vs 4.1%, aOR 3.27, 95%CI 2.67–4.00) were increased in the GC group compared to the non-GC group (both, *P* < 0.001). On the contrary, odds of cesarean delivery (23.6% vs 31.6%, aOR 0.59, 95%CI 0.51–0.69) were decreased in the GC group compared to the non-GC group among singleton pregnancies (both, *P* < 0.001). There was interaction per the extent of gestation, and these outcome measures for GC compared to non-GC were either statistically not significant or less robust among multi-fetal gestations (Table 4).

**Table 4:** Delivery outcomes stratified by fetal number

Characteristic	Singleton				Multifetal gestations			
	non-GC	GC	aOR (95%CI)	*P*-value	non-GC	GC	aOR (95%CI)	*P*-value
**Gestational age (w)**								
≥ 39	62.4	61.8	1.00 (reference)		2.4	0	n/a	
37–38	27.1	23.8	0.93 (0.79–1.09)	0.375	35.3	55.3	1.83 (1.14–2.95)	0.012
34–36	6.4	10.8	1.79 (1.44–2.23)	< 0.001	38.4	27.6	0.87 (0.52–1.45)	0.580
26–33	2.1	2.3	1.07 (0.68–1.70)	0.764	18.0	15.8	1.00 (reference)	
22–25	0.4	1.1	2.54 (1.32–4.89)	< 0.001	2.8	0	n/a	
< 22	0.3	0	n/a		1.7	0	n/a	
Unknown	1.3	**	0.18 (0.05–0.73)	0.016	1.4	**	0.97 (0.19–4.87)	0.970
**Cesarean delivery**								
No	68.4	76.4	1.00 (reference)		26.4	32.9	1.00 (reference)	
Yes	31.6	23.6	0.59 (0.51–0.69)	< 0.001	73.6	67.1	0.73 (0.52–1.02)	0.077
**Primary cesarean delivery**								
No	80.6	88.1	1.00 (reference)		31.2	39.2	1.00 (reference)	
Yes	19.4	11.9	0.47 (0.38–0.59)	< 0.001	68.8	60.8	0.64 (0.42–0.87)	0.007
**Vaginal birth after cesarean**								
No	86.8	85.4	1.00 (reference)		95.2	**	1.00 (reference)	
Yes	13.2	14.6	1.13 (0.71–1.79)	0.631	4.8	**	0.69 (0.10–4.81)	0.708
**Operative delivery**^§^								
No	94.3	94.2	1.00 (reference)		89.3	**	1.00 (reference)	
Yes	5.7	5.8	1.02 (0.74–1.41)	0.878	10.7	**	0.14 (0.02–1.09)	0.060
**Postpartum hemorrhage**								
No	95.9	87.8	1.00 (reference)		89.9	84.2	1.00 (reference)	
Yes	4.1	12.2	3.27 (2.67–4.00)	< 0.001	10.1	15.8	1.55 (1.00–2.40)	0.051
**Blood transfusion**								
No	98.9	98.9	1.00 (reference)		95.5	**	1.00 (reference)	
Yes	1.1	1.1	1.02 (0.55–1.90)	0.872	4.5	**	1.50 (0.77–2.85)	0.233
**Severe maternal morbidity**^†^								
No	98.2	98.9	1.00 (reference)		93.7	**	1.00 (reference)	
Yes	1.8	1.1	0.62 (0.33–1.16)	0.162	6.3	**	1.05 (0.55–1.99)	0.867
**Hysterectomy**								
No	99.8	**	1.00 (reference)		99.5	100	1.00 (reference)	
Yes	0.2	**	0.50 (0.07–3.57)	0.999	0.5	0	n/a	0.999

Percentage per group is shown. The analytic approach followed the main cohort in each stratum. **A small number of data were suppressed per HCUP guidelines. ^§^Vaginal delivery. ^†^Including blood transfusion. Abbreviations: *GC*, gestational carrier; *aOR*, adjusted odds ratio; *CI*, confidence interval

## Discussion

### Principal findings

The key findings of this study include the gradual increase in GC pregnancies over time and further insight into GC pregnancies and the associated mixed effects for obstetrical complications and adverse delivery outcomes. The reason for an increased risk of adverse outcomes in GC pregnancies needs further exploration. Prior research has pointed to the increased risks associated with the use of ART. A retrospective cohort study comparing a patient’s own non-ART pregnancy to an ART pregnancy in which they were a GC found increased odds of placenta previa, gestational hypertension, preterm birth, and low birth weight in the GC pregnancy [6]. As such, it is likely that the increased pregnancy risks for a GC are due, at least in part, to the ART pregnancy in and of itself. Inherent maternal risk factors likely also contribute but are harder to quantify. Moreover, there are numerous ethical discussions that must be considered with GC pregnancies. People who consider serving as a GC should have a robust amount of data available to fully evaluate the risk profile associated with this unique type of pregnancy.

### Insights from results

#### Gestational carrier trends and characteristics

Our study found the prevalence of GC pregnancies increased significantly by 55% throughout the study period. The increase in GC pregnancies is consistent with prior studies [3, 4]. The percentage of ART cycles attributable to GCs has increased from 1 to 5.4% in 2019 [19]. Reasons for the increase in use include growing demand from higher infertility rates, increased awareness of assisted reproductive technology (ART), and utilization of services by same-sex couples. Commercialization of GC pregnancies and the use of marketing is also likely to have contributed [20, 21]. Additionally, an increasing number of states are passing legislation which supports GCs, and international intended parents are also utilizing gestational surrogacy in the U.S. as it is still illegal in many countries. [22]

#### Maternal obstetric outcomes

The rate of multifetal pregnancies (13.7%) in our data is similar to the multifetal gestation rate from the Society of Assisted Reproductive Technology database. A review of this data from 2009 to 2013 found GC pregnancies compared to non-GC IVF pregnancies result in higher order multiples, 14.8% and 12.6% of the time, respectively. [4] They occur almost entirely (90%) after the transfer of multiple embryos, a practice no longer supported by the ASRM. A prior study specifically examining GC carriers found an association between adverse perinatal outcomes and the transfer of multiple embryos [23]. Importantly, our study did find a significant decrease (70.4%) in multiple gestation pregnancies noted for GC carriers over the study period. This may signify increased awareness of the risks associated with multiple pregnancies and adherence to newer ASRM guidelines calling for single embryo transfers almost exclusively. [10]

Preterm premature rupture of membranes occurred in 5.4% of GC pregnancies compared to 2.7% of the non-GC pregnancies in our study. Preterm premature rupture of the membrane is one of the most common reasons for antepartum hospitalization, and antepartum hospitalization has been linked to adverse obstetrical outcomes [24]. Current guidelines in the United States recommend hospitalization until delivery, and prolonging the time until delivery is primarily for fetal benefit [25]. Importantly, hospitalization may be necessary for several weeks and result in significant disruptions to daily life for GCs. Notably, chorioamnionitis occurred significantly less among GC carrier pregnancies as compared to non-GC carrier pregnancies in this study, warranting further evaluation.

We also noted several placental disorders to be associated with GC pregnancies, including placental abruption and low-lying placenta. This is likely related to the use of ART in GC pregnancies, as it is a known risk factor for abnormal placentation [26–30]. The reasons for abnormal placentation in ART pregnancies are still not fully understood. [31]

#### Delivery outcomes

The majority of GC carrier pregnancies were delivered at term, but pregnant patients in the GC group were more likely to have late-preterm delivery (13.2%) compared to those in the non-GC group (7.0%). Various studies have found an elevated risk of preterm birth in IVF pregnancies, regardless of the number of gestations [32–36]. In a 2016 retrospective cohort study of data from the Centers for Disease Control and Prevention’s National ART Surveillance System from 2009 to 2013, authors found that for fresh donor oocyte cycles, GC pregnancies resulted in preterm birth in 32.7% of deliveries which was similar to non-GC pregnancies. When the groups were stratified by plurality, they noted singleton pregnancies were less likely to result in preterm birth compared to non-GC pregnancies.

The GC pregnancy was associated with a nearly threefold increased risk of postpartum hemorrhage and this association persists when comparing singleton-only pregnancies. This may be a byproduct of certain characteristics of the IVF process. Given the nature of GC pregnancies and the risk of a spontaneous pregnancy in these individuals, if attempting a natural cycle frozen embryo transfer, programmed frozen embryo transfer is far more common. There is growing literature on the increased risks of a programmed cycle frozen embryo transfer compared to natural cycle frozen embryo transfer and one of these potential risks includes postpartum hemorrhage [37]. Serious complications resulting from postpartum hemorrhage include blood transfusion, peripartum hysterectomy, and overall increased maternal morbidity [38–42]. Prospective GCs need to be counseled on these potential risks.

Both the overall and the primary cesarean delivery rates for the GC group were lower than the non-GC group when comparing singleton pregnancies. One factor to consider is the lower rate of obesity in the GC group, as this decreases the chance of cesarean delivery. Furthermore, ASRM guidelines recommend comprehensive evaluation of anyone who wishes to be a gestational carrier, and this promotes the selection of overall healthy individuals. There are specific guidelines to select individuals with a history of successful term delivery and a low number of cesarean sections [1]. This practice may result in lower rates of cesarean delivery for the GC group. This is supported by a 2022 cross-sectional study comparing outcomes for 361 GC deliveries in Utah from 2009 to 2018 which found higher rates of cesarean delivery among women who did not meet ASRM guidelines in comparison to those who meet guidelines (36.2% vs 23.4%, OR 1.85, 95% CI 1.02–3.37). [7]

We found no increase in maternal morbidity associated with GC pregnancy. Similarly, a prior study comparing GC and non-GC IVF pregnancies found that severe obstetric morbidity was uncommon in GC pregnancies; however, different definitions of morbidity were used. [43]

### Study limitations

This study is limited by the fact that it is a retrospective data analysis. Thus, this data may contain possible unmeasured confounders and can only describe associations between information, not establish causation, all of which are general limitations of all large database publications. Given the reliance on ICD-10 coding by physicians, the increasing rates of GC pregnancies may be skewed due to increased physician awareness and use of the code for GC pregnancies as time goes on, as opposed to an actual increase in the number of GC pregnancies. This data is also unable to account for individual patient obstetrics history or IVF cycle and treatment data which was not included within the database. The accuracy of GC pregnancies was not assessable without medical record review in this study. Lastly, this data does not consider the short- and long-term neonatal outcomes of GC pregnancies. Research has been conducted to show the differences in neonatal outcomes comparing GC pregnancies and non-ART pregnancies [6], but further research should be done to evaluate the differences between GC and non-GC ART pregnancies.

## Conclusion

We found mixed obstetric outcomes in singleton GC pregnancies. Several adverse obstetric and delivery outcomes are noted to be increased among pregnant GCs whereas cesarean delivery rates are lower. These increased risks are likely related to ART use and rates of multifetal gestations. Even so, further exploration in a prospective sample of patients is warranted. Future studies should explore the risks to GCs based on various ART cycle characteristics. This data provides additional evidence that can be useful for counseling prospective GCs on the risks associated with GC pregnancies.

Meeting presentation: ASRM 2023 Scientific Congress & Expo New Orleans, LA, October 14–18, 2023.

## Supplementary Information

Below is the link to the electronic supplementary material.

Supplementary file1 (DOCX 55 KB)

## Data Availability

The data on which this study is based are publicly available upon request at the Healthcare Cost and Utilization Project, Agency for Healthcare Research and Quality, https://www.hcup-us.ahrq.gov/nisoverview.jsp.

## Declarations

### Attestation statements

The manuscript’s corresponding authors affirm that the manuscript is an honest, accurate, and transparent account of the study being reported, that no important aspects of the study have been omitted, and that any discrepancies from the study as planned (and, if relevant, registered) have been explained. The National Inpatient Sample is developed for the Healthcare Cost and Utilization Project that is sponsored by the Agency for Healthcare Research and Quality, and the program is the source of the de-identified data used; race/ethnicity was grouped by the program; and the program has not verified and is not responsible for the statistical validity of the data analysis or the conclusions derived by the study team.
