# Supplementary material for: National-level assessment of gestational carrier pregnancies in the United States
Source: J Assist Reprod Genet. 2024 Nov 20;42(1):201–11. doi: 10.1007/s10815-024-03320-5 (PMC11805738; doi:10.1007/s10815-024-03320-5)
Supplement: Supplementary file 1 — Supplementary file1 (DOCX 55 KB) [file 10815_2024_3320_MOESM1_ESM.docx]

**Supplemental Online Content**

National-level assessment of gestational carrier pregnancies in the United States.

**Supplemental Table S1.** Coding information.

**Supplemental Table S2.** Standardized difference for IPTW analysis.

This supplemental material has been provided by the authors to give readers additional information about their work.

**Supplemental Table S1. Coding information.**

|  | **Diagnosis-Related Group Codes** | **ICD-10 CM Codes** | **ICD-10 PCS Codes** |
| --- | --- | --- | --- |
| Gestational carrier |  | Z333 |  |
| Vaginal delivery | 767, 768, 774, 775, 796, 797, 798, 805, 806, 807 | O80 | 10D07Z3, 10D07Z4, 10D07Z5, 10D07Z6, 10D07Z7, 10D07Z8 |
| Cesarean delivery | 765, 766, 783, 784, 785, 786, 787, 788 | O82, O7582 | 10D00Z0, 10D00Z1, 10D00Z2 |
| Obesity |  | E660, E661, E662, E668, E669, Z683, Z684, O9921 |  |
| Asthma |  | J45 |  |
| Hypertensive disorder |  | O10, O11, O13, O14, O15, O16 |  |
| Diabetes mellitus |  | O24 |  |
| Unhoused status |  | Z590 |  |
| Tobacco use |  | Z720, O9933, F17, T652 |  |
| Alcohol use |  | F10, O9931, T51, Q860 |  |
| Illicit drug use |  | F11, F12, F13, F14, F15, F16, F18, F190, O9932 |  |
| Schizophrenia disorder |  | F20, F25 |  |
| Bipolar disorder |  | F31, F30 |  |
| Depressive disorder |  | F33, F32 |  |
| Anxiety disorder |  | F41 |  |
| Gonorrhea |  | O982 |  |
| Syphilis |  | O981 |  |
| Hepatitis virus |  | O984 |  |
| Herpes |  | A60 |  |
| Prior uterine scar |  | O342 |  |
| Uterine myoma |  | D25, O341 |  |
| Uterine adenomyosis |  | N800 |  |
| Uterine anomaly |  | Q51, O340 |  |
| Endometriosis |  | N80 |  |
| Polycystic ovary synd |  | E282 |  |
| Carcinoma in-situ |  | D06 |  |
| Grand multiparity |  | O094, Z641 |  |
| Prior pregnancy losses |  | O262 |  |
| Excess gaining weight |  | O260 |  |
| Gestational age |  | Z3A |  |
| Placenta previa |  | O44 |  |
| Low-lying placenta |  | O444, O445 |  |
| Placenta abruption |  | O45 |  |
| Placenta accreta spectrum |  | O432 |  |
| Placenta malformation |  | O431 |  |
| Vasa previa |  | O694 |  |
| Uterine rupture |  | O71 |  |
| Cervical insufficiency |  | O343 |  |
| Fetal growth restriction |  | O365, Z364 |  |
| Large for gestational age |  | O366 |  |
| Multifetal gestation |  | O30, O31, O632, O661, Z372, Z373, Z374, Z375, Z376, Z377, O4302 |  |
| Fetal breech position |  | O321, O641 |  |
| Umbilical cord prolapse |  | O690 |  |
| Fetal anomaly |  | O35 |  |
| Fetal demise |  | O364 |  |
| Polyhydramnios |  | O40 |  |
| Oligohydramnios |  | O410 |  |
| PROM |  | O42 |  |
| Chorioamnionitis |  | O411 |  |
| Postpartum hemorrhage |  | O72, R58 |  |
| Blood products transfusion |  |  | ** |
| Sever maternal morbidity |  | ** | ** |

** Per Centers for Disease and Control Prevention definition ( https://www.cdc.gov/reproductivehealth/maternalinfanthealth/smm/severe-morbidity-ICD.htm)

**Supplementary Table S2. Standardized difference for IPTW analysis.**

| Characteristic | Pre | Post |
| --- | --- | --- |
| Primary payer | 1.042 | 0.696 |
| Hospital region | 0.850 | 0.159 |
| Patient age | 0.631 | 0.461 |
| Race and ethnicity | 0.462 | 0.439 |
| Tobacco use | 0.261 | 0.272 |
| Year | 0.228 | 0.115 |
| Drug use | 0.210 | 0.134 |
| Obesity | 0.198 | 0.034 |
| Hospital bed capacity | 0.161 | 0.114 |
| Hospital teaching setting | 0.124 | 0.092 |
| Pregestational hypertension | 0.111 | 0.036 |
| Depressive disorder | 0.047 | 0.027 |
